# Oral curcumin for Alzheimer's disease: tolerability and efficacy in a 24-week randomized, double blind, placebo-controlled study

**DOI:** 10.1186/alzrt146

**Published:** 2012-10-29

**Authors:** John M Ringman, Sally A Frautschy, Edmond Teng, Aynun N Begum, Jenny Bardens, Maryam Beigi, Karen H Gylys, Vladimir Badmaev, Dennis D Heath, Liana G Apostolova, Verna Porter, Zeba Vanek, Gad A Marshall, Gerhard Hellemann, Catherine Sugar, Donna L Masterman, Thomas J Montine, Jeffrey L Cummings, Greg M Cole

**Affiliations:** 1 Mary S. Easton Center for Alzheimer's Disease Research, David Geffen School of Medicine at UCLA, 10911 Weyburn Ave., #200, Los Angeles, CA 90095-7226, USA; 2 UCLA Department of Neurology, David Geffen School of Medicine at UCLA, 710 Westwood Plaza, Los Angeles, CA 90095-1769, USA; 3 UCLA Department of Medicine, 757 Westwood Plaza, Los Angeles, CA 90095, USA; 4 Geriatric Research Education and Clinical Center, W LA Veterans Administration Medical Center, 11301 Wilshire Blvd., Bldg. 113, Room 312, Los Angeles, CA 90073, USA; 5 UCLA School of Nursing, Box 956919, 6-2668 Factor Bldg., Los Angeles, CA 90095-6919, USA; 6 AMH Corporation, 1440-6 Forest Hill Road, Staten Island, NY 10314, USA; 7 Moores UCSD Cancer Center, 3855 Health Sciences Drive, La Jolla, CA 92093-0901, USA; 8 Center for Alzheimer Research and Treatment, Harvard Medical School, 221 Longwood Avenue, Boston, MA 02115, USA; 9 UCLA Semel Institute for Psychiatry and Human Behavior, Box 951759, C9-432 Semel, Los Angeles, CA 90095-1759, USA; 10 F. Hoffman-La Roche, Ltd, Grenzacherstrasse 183, Bldg/Room 74/3W.306B, 4070 Basel, Switzerland; 11 Department of Neuropathology, University of Washington Medical Center, Room C-516, Box 357470, Seattle, WA 98195, USA; 12 Cleveland Clinic Lou Ruvo Center for Brain Health, 888 W. Bonneville, Las Vegas, NV 89106, USA

## Abstract

**Introduction:**

Curcumin is a polyphenolic compound derived from the plant *Curcuma Long Lin *that has been demonstrated to have antioxidant and anti-inflammatory effects as well as effects on reducing beta-amyloid aggregation. It reduces pathology in transgenic models of Alzheimer's disease (AD) and is a promising candidate for treating human AD. The purpose of the current study is to generate tolerability and preliminary clinical and biomarker efficacy data on curcumin in persons with AD.

**Methods:**

We performed a 24-week randomized, double blind, placebo-controlled study of Curcumin C3 Complex^® ^with an open-label extension to 48 weeks. Thirty-six persons with mild-to-moderate AD were randomized to receive placebo, 2 grams/day, or 4 grams/day of oral curcumin for 24 weeks. For weeks 24 through 48, subjects that were receiving curcumin continued with the same dose, while subjects previously receiving placebo were randomized in a 1:1 ratio to 2 grams/day or 4 grams/day. The primary outcome measures were incidence of adverse events, changes in clinical laboratory tests and the Alzheimer's Disease Assessment Scale - Cognitive Subscale (ADAS-Cog) at 24 weeks in those completing the study. Secondary outcome measures included the Neuropsychiatric Inventory (NPI), the Alzheimer's Disease Cooperative Study - Activities of Daily Living (ADCS-ADL) scale, levels of Aβ_1-40 _and Aβ_1-42 _in plasma and levels of Aβ_1-42_, t-tau, p-tau_181 _and F2-isoprostanes in cerebrospinal fluid. Plasma levels of curcumin and its metabolites up to four hours after drug administration were also measured.

**Results:**

Mean age of completers (n = 30) was 73.5 years and mean Mini-Mental Status Examination (MMSE) score was 22.5. One subject withdrew in the placebo (8%, worsened memory) and 5/24 subjects withdrew in the curcumin group (21%, 3 due to gastrointestinal symptoms). Curcumin C3 Complex^® ^was associated with lowered hematocrit and increased glucose levels that were clinically insignificant. There were no differences between treatment groups in clinical or biomarker efficacy measures. The levels of native curcumin measured in plasma were low (7.32 ng/mL).

**Conclusions:**

Curcumin was generally well-tolerated although three subjects on curcumin withdrew due to gastrointestinal symptoms. We were unable to demonstrate clinical or biochemical evidence of efficacy of Curcumin C3 Complex^® ^in AD in this 24-week placebo-controlled trial although preliminary data suggest limited bioavailability of this compound.

**Trial registration:**

ClinicalTrials.gov Identifier: NCT00099710.

## Introduction

More than five million Americans currently have Alzheimer's disease (AD) [1] and available treatments have only modest symptomatic benefits. We are in desperate need of readily available, safe interventions that modify disease course. Curcumin is a polyphenolic compound derived from the plant *Curcuma Long Lin *that has been used as a food preservative and to treat various ailments in Ayurvedic medicine. In vitro and in vivo animal studies have demonstrated that curcumin has potentially important biological effects including anti-oxidant and anti-inflammatory properties [2]. As oxidative stress [3] and inflammation [4] are potentially involved in propagating AD pathology, the utility of curcumin in treating and preventing AD is being pursued [5].

There is convergent evidence that overproduction, aberrant aggregation, or decreased elimination of different forms of amyloid beta protein (Aβ) are critical events in the pathogenesis of AD and, therefore, treatment development has focused on these processes. Curcumin has a biphenolic structure similar to Congo Red and binds to amyloid plaques *in vivo *[6]. Curcumin prevents Aβ-induced toxicity *in vitro *in various preparations [7] and prevented the aggregation of Aβ into fibrils [8]. Transgenic mice harboring the amyloid precursor protein Swedish mutation (APPsw) fed curcumin for six months had decreased levels of Aβ and inflammatory cytokines in the brain [9]. The ability of curcumin to cross the blood-brain barrier, and bind to and induce rapid dissolution of plaques was verified using multi-photon microscopy *in vivo *in APPsw/PS1dE9 mice [10]. In further studies of curcumin and tetrahydrocurcumin in the Tg2576 APP*sw *mouse, curcumin was found to reduce central nervous system levels of IL-1β and isoprostanes (an index of oxidative stress) and to reduce amyloid plaque burden [6].

Although there is extensive experience with human consumption of turmeric oleoresin (85% curcumin) such that it is classified as 'Generally Recognized As Safe' (GRAS) by the US Food and Drug Administration, there is still much to be determined about the tolerability and bioavailability of moderate doses of curcumin when used chronically, particularly in elderly individuals. Prior short-term human studies suggest the potential for gastrointestinal side effects including diarrhea [11] and toxicity studies in rats indicate the possibility of hepatoxicity and thyroid follicular cell hyperplasia [12]. We, therefore, performed a 24-week, randomized, double-blind, placebo-controlled study of two doses of Curcumin C3 Complex^® ^in persons with mild-to-moderate AD with an open-label extension to 48 weeks. The primary goals were to gather data on safety and tolerability and preliminary data on efficacy with regard to cognition. Secondary goals were to obtain preliminary efficacy data on behavior and activities of daily living and on plasma and cerebrospinal fluid (CSF) AD biomarkers.

## Materials and methods

### Participants

Thirty-six subjects with mild-to-moderate probable AD were enrolled at the Mary S. Easton Center for Alzheimer's Disease Research at UCLA. Inclusion criteria were the presence of dementia according to the Diagnostic and Statistical Manual of Mental Disorders, fourth edition (DSM-IV) [13], a diagnosis of probable AD by the National Institute of Neurological and Communicative Disorders and Stroke - Alzheimer's Disease and Related Disorders Association (NINCDS-ADRDA) criteria [14], age greater than 49 years, Mini-Mental Status Examination (MMSE) scores between 17 and 29, English proficiency, and the availability of a study partner to monitor medication administration and adverse effects. Dosage of acetylcholinesterase inhibitors (AchE-I) and memantine had to be stable for one month prior to enrollment. Exclusion criteria included significant systemic illness or recent history of gastrointestinal bleeding. Exclusionary medications included aspirin at doses greater than 325 mg/day, use of non-steroidal anti-inflammatory drugs more than three times a week, coumadin, heparin, gingko biloba, and antioxidant supplements (for example, coenzyme Q_10 _and alpha-lipoic acid). Concomitant consumption of vitamin E at doses up to 2,000 i.u./day and vitamin C up to 500 mg/day were allowed. To the extent possible, doses of medications and supplements were stable throughout the course of the trial.

A single block of 36 sequential assignments was randomly created and the UCLA research pharmacy packaged and distributed the investigational product accordingly. All study personnel and subjects were blinded to randomization sequence. An unblinded data safety and monitoring board met by teleconference before, and on three occasions during, the study. Subjects or their legally authorized representatives provided written informed consent prior to the initiation of any study procedures. The protocol was approved by the UCLA Institutional Review Board.

#### Intervention

Subjects were allocated to receive placebo, 2 gm, or 4 gm of Curcumin C3 Complex^® ^per day in two divided doses for 24 weeks in a 1:1:1 ratio. These doses were chosen based on extrapolation from animal studies and allometric scaling. Curcumin C3 Complex^® ^is an orange-yellow crystalline powder plant extract that was encapsulated and provided (as was the matching placebo) by Sabinsa Corporation (Piscataway, NJ, USA). Curcumin C3 Complex^® ^consists of 95% curcuminoids with 70% to 80% comprised by curcumin, 15% to 25% demethoxycurcumin, and 2.5% to 6.5% bisdemethoxycurcumin. This content was independently verified using HPLC by author DDH. Curcumin C3 Complex^® ^or placebo was administered orally as four 500 mg capsules taken twice daily with a fatty meal. At the 24-week visit, all subjects receiving placebo were randomized to one of the two doses of curcumin that was administered for another 24 weeks. Subjects receiving 2 grams or 4 grams of curcumin in the first 24 weeks continued to 48 weeks on the same dose.

### Outcome measures

Primary outcomes included tolerability and cognitive measures. At baseline and each of the post-baseline visits (weeks 4, 12, 24, 36, and 48) subjects and caregivers were interviewed regarding adverse events using a checklist. At screening and at each post-baseline visit, vital signs were taken and laboratory value monitoring performed including complete blood count, chemistry panel, lipid profile, thyroid stimulating hormone and thyroxine levels. Bleeding times before and after treatment were also obtained on 24 subjects. The Alzheimer's Disease Assessment Scale, cognitive sub-portion (ADAS-Cog) [15] was administered at baseline and at the 24 and 48 week visits. The current paper describes the results of efficacy outcome measures at 24 weeks and tolerability measures out to 48 weeks.

Secondary clinical outcome measures included the Neuropsychiatric Inventory (NPI) [16] and the Alzheimer's Disease Cooperative Study Activities of Daily Living (ADCS-ADL) [17] instrument which were administered to caregivers at baseline and at the 24 and 48 week visits. The MMSE was also administered at screening and at all post-baseline visits. Plasma and CSF were collected at the baseline visit for biomarker measurements; this was repeated at week 24. All samples were centrifuged and frozen at -80°C. Commercially available kits were used to measure plasma levels of Aβ_1-40 _and Aβ_1-42 _(INNO-BIA Plasma Aβ Forms; Innogenetics, Ghent, Belgium) and CSF levels of Aβ_1-42_, total tau (t-tau), and tau phosphorylated at threonine 181 (p-tau_181_, INNO-BIA AlzBio3; Innogenetics, Ghent, Belgium) using the Luminex xMAP platform (Luminex, Austin TX, USA). CSF isoprostanes (F_2_-IsoPs) were quantified using a stable isotope dilution assay with gas chromatography/mass spectrometry and selective ion monitoring as described previously [18]. All plasma and CSF assays were done in a single batch at the end of the 24-week study period.

### Pharmacokinetic analysis

Pharmacokinetic analyses were performed in two different manners in two different laboratories.

First, plasma levels of native curcumin at baseline and at 0.5, 1, 2, 3, and 4 hours post-dose at the 24 week visit were measured using HPLC as described previously [19]. The coefficient of variation (CV) for a 20 ng/mL sample was 4.95%. The lower limit of detection using HPLC was 200 ng/mL.

Second, using liquid chromatography/tandem mass spectrometry (LC/MS/MS) plasma (baseline levels and 3 hour post-dose at the 24 week visit) and CSF levels (prior to the first dose and after the dose at the 24 week visit) of native curcumin were measured. Curcumin was extracted from plasma and CSF in 95% ethyl acetate/5% methanol and analyzed by LC/MS/MS, using tetramethoxycurcumin as an internal standard as previously described [6]. The LC/MS/MS system (Varian International, Lake Forest, CA, USA) consisted of the proStar 410 autosampler and pumps and a 310 mass spectrometer with ESI (-) which is able to optimize each ion automatically. Dried plasma/CSF extract samples were re-dissolved in acetonitrile:water (50/50, by volume) in 10 mM ammonium acetate, and an aliquot of the solution (typically 50 ul) was injected onto a reverse phase HPLC column (Atlantis T3, 150 × 2.1 mm; Waters Corporation, Milford, MA, USA). The *m*/*z *values of the specific transitions were for curcumin, 367.0, 148.7, 172.4, 216.3, tetrahydrocurcumin, 372.0, 193.5, 236.0 and for tetramethoxycurcumin, 396.4, 149.5, 297.1, 337.6 (the internal standard). Instrument manufacturer-supplied LC/MS/MS Varian software (Varian MS Workstation, Version 6.9.1) was used to calculate each ion according to its qualifier ion. The limit of detection for curcumin and tetrahydrocurcumin in the plasma sample was 1 and 3 ng/mL, respectively. To measure glucuronides, 10 μL of 0.4 M PBS with 2% ascorbic acid and 0.1% ethylenediaminetetraacetic acid (EDTA) (pH 3.6) were added to 0.5 ml plasma along with 30 μL of 0.1 M PBS (pH 6.8). Then, 500 Fishman units of β-glucuronidase (Sigma, St Louis, MO, USA) were added to the sample, which was incubated at 37°C for 45 minutes at pH 5.0, followed by analysis of curcumin and tetrahydrocurcumin using LC/MS/MS.

### Statistical analysis

The pre-specified data analysis plan for efficacy measures was repeated measures analyses of variance (ANOVAs) with drug dose as the independent variable and cognitive, behavioral, and biomarker measures as the dependent variables over time. Occurrence of adverse events, changes in vital signs, and safety laboratory results per subject according to the treatment they received over the course of the study were compared between treatment groups by the general linear mixed model.

Analyses of efficacy measures were performed on data from completers due to lack of follow-up biomarker data for subjects who discontinued. Analyses of safety and tolerability measures were performed on all available data from all available subjects. The funding organizations had no role in study design or outcome analysis.

## Results

**Figure 1:**
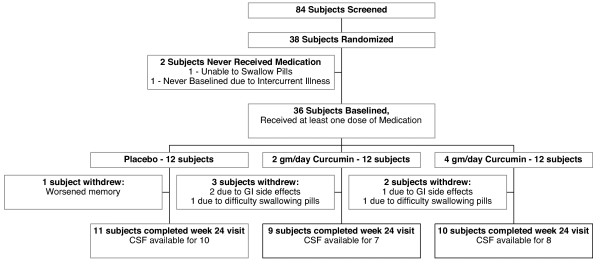
**Subject participation flow chart**.

Eighty-four subjects were screened between August 2004 and January 2007 of whom 38 were randomized (Figure 1). Two subjects never received study medication (one due to inability to swallow the capsules, another due to intercurrent illness between randomization and baseline visits) and their treatment allocations were re-assigned. Thirty-six subjects therefore received at least one dose of study medication (12 per group). Among the 30 subjects who completed 24 weeks, baseline gender (63% female), years of education (15.2 years), use of AchE-Is (93%) and memantine (77%), average age (73.5 years), MMSE (22.5), total NPI (9.7), ADAS-Cog (19.4), and ADCS-ADL (62.8) scores did not differ among treatment groups (Table 1).

**Table 1:** Baseline characteristics of subjects who completed the 24-week, double blind, placebo controlled portion of the study.

	Placebo (n = 11)	Curcumin 2 gm/day (n = 9)	Curcumin 4 gm/day (n = 10)	
Age in years	70.2 (12.4)	76.7 (5.6)	75.3 (6.9)	*P *= 0.71
Gender, number of women (%)	6 (55%)	6 (67%)	7 (70%)	*P *= 0.74
Years of education (S.D.)	14.3 (2.5)	16.7 (1.3)	15.0 (2.7)	*P = 0.08*
Number on AchE-I's (%)	11 (100%)	*7 (78%)*	10 (100%)	*P = 0.08*
Number on memantine (%)	8 (73%)	7 (78%)	8 (80%)	*P *= 0.92
MMSE (S.D., range)	23.2 (2.3, 20-27)	21.4 (3.2, 17-28)	22.8 (3.4, 18-27)	*P *= 0.41
ADAS-Cog score (S.D.)	18.2 (4.9)	20.9 (7.4)	19.3 (3.4)	*P *= 0.64
NPI total score (S.D.)	8.5 (12.4)	8.9 (9.6)	11.7 (16.9)	*P *= 0.84
ADCS-ADL score (S.D.)	66.7 (3.6) (n = 10)	60.3 (10.9)	61.0 (8.4)	*P *= 0.18

Note that ADCS-ADL score was not available on one subject in the placebo arm. AchE-I, acetylcholinesterase inhibitor; ADAS-Cog, Alzheimer's Disease Assessment Scale - Cognitive Subscale; ADCS-ADL, Alzheimer's Disease Cooperative Study - Activities of Daily Living; MMSE, Mini-Mental State Examination; NPI, Neuropsychiatric Inventory; S.D., standard deviation.

Between baseline and 24 weeks, six subjects withdrew. One subject (8%) withdrew in the placebo group (worsened memory) and 5/24 subjects (21%) in the curcumin group withdrew due to adverse effects (*P *= 0.33). Among the curcumin-treated subjects, three withdrew in the 2 gm/day curcumin group (two due to gastrointestinal side effects of black stools and diarrhea, one due to subsequent difficulty swallowing the pills), and two subjects withdrew in the 4 gm/day curcumin group (one due to diarrhea and one due to difficulty swallowing the pills). Of the 30 subjects entering the open-label phase, two subjects originally assigned to placebo dropped out before week 48, one due to deterioration of AD symptoms and one due to weight loss and a low hematocrit.

Overall, adverse events occurred in 91.7% of placebo-treated subjects and 100% of curcumin treated subjects (n.s.). Complaints attributable to the endocrine system were less common in the 2 gm/day curcumin group, occurring at 3% of visits, compared to the placebo group (17%) and the 4 gm/day group (19%, *P *= 0.02). Patients on placebo reported diarrhea on 4% of visits, patients on 2 gm/day of curcumin at 6% of visits and patients on 4 gm/day on 8% of visits (*P *= 0.63). Joint pain was reported at 15% of visits for persons on placebo, 9% of visits for persons on 2 gm and 5% of persons on 4 gm of curcumin (*P *= 0.19). When modeling the difference in joint pain occurrence as a linear function of curcumin dose, a 2.5% trend towards decrease in joint pain per gram of curcumin was found (*P *= 0.07). There were no serious adverse events in the course of this study.

Complete safety laboratory data at baseline and 24 weeks were available for 11 completers on placebo, 9 on 2 gm/day and 9 on 4 gm/day of curcumin. Controlling for baseline values, subjects on curcumin had statistically lower hematocrit (placebo = 42.9%, standard error of the mean (SEM) = 0.49, 2 gm = 41.8%, SEM = 0.55, 4 gm = 42.3%, SEM. = 0.54; *P *= 0.014) and higher glucose levels (placebo = 85.9 mg/dL, SEM = 4.2, 2 gm = 91.6 mg/dL, SEM = 4.5; 4 gm = 90.5 mg/dL, SEM = 4.5, *P *= 0.043) while being treated.

There were no differences between treatment groups in change in ADAS-Cog, NPI, ADCS-ADL, or MMSE scores using repeated measures ANOVA (Table 2). When the 2 gm/day and 4 gm/day curcumin groups were combined, there was a trend for treated subjects to do worse than placebo-treated subjects on the MMSE (*P *= 0.08). Pre- and post-treatment CSF was available for ten completers in the placebo group, seven in the 2 gm/day group, and for eight in the 4 gm/day group. There were no significant effects of treatment group on change in plasma Aβ_1-40 _and Aβ_1-42_, CSF Aβ_1-42_, CSF tau or p-tau or F_2_-IsoPs (Table 2).

**Table 2:** Mean baseline, week 24, and change in clinical and biochemical measures.

	Placebo (n = 11)			2 gm/day Curcumin (n = 9)			4 gm/day Curcumin (n = 10)			
	
	baseline	week 24	change	baseline	week 24	change	baseline	week 24	change	
MMSE	23.2 (2.3)	22.7 (2.3)	-0.45 (2.6)	21.4 (3.2)	19.6 (3.9)	- 1.89 (2.6)	22.8 (3.4)	19.9 (3.0)	-2.90 (2.0)	*P *= 0.08
ADAS-Cog	18.2 (4.9)	20.4 (7.1)	2.2 (5.0)	20.9 (7.4)	27.2 (11.7)	6.3 (8.1)	19.3 (3.4)	22.4 (4.4)	3.1 (3.4)	*P *= 0.26
NPI total	8.5 (12.4)	10.3 (14.3)	1.8 (10.0)	8.9 (9.6)	11.0 (15.8)	2.1 (10.1)	11.7 (16.9)	6.9 (5.6)	-4.8 (14.7)	*P *= 0.35
ADCS-ADL	66.7 (3.6)	60.6 (9.5)	-4.9 (7.6)	60.3 (10.9)	53.1 (15.4)	-6.3 (6.8)	61.0 (8.4)	56.2 (9.4)	-3.4 (6.5)	*P *= 0.72
Plasma Aβ_42 _(pg/mL)	28.7 (11.2)	31.4 (13.5)	2.7 (4.5)	25.3 (8.2)	25.3 (10.4)	0.0 (3.3)	29.6 (4.8)	30.5 (6.7)	0.9 (3.5)	*P *= 0.98
Plasma Aβ_40 _(pg/mL)	234.2 (60.3)	220.4 (74.9)	-13.8 (64.9)	239.4 (41.8)	226.1 (27.3)	-13.3 (39.6)	224.9 (38.5)	219.4 (57.6)	-18.9 (58.1)	*P *= 0.29
CSF Aβ_42 _(pg/mL)	150.7 (26.2)	157.5 (32.0)	6.8 (19.8)	150.2 (35.0)	159.2 (30.8)	9.0 (17.0)	159.1 (27.2)	168.8 (30. 7)	9.7 (30.8)	*P *= 0.96
CSF Total tau (pg/mL)	110.9 (75.9)	114.7 (82.9)	3.8 (27.9)	95.8 (31.5)	95.9 (23.2)	0.1 (14.5)	146.0 (78.0)	149.3 (81.1)	3.3 (38.3))	*P *= 0.97
CSF Phospho-tau (pg/mL)	48.6 (18.8)	51.3 (19.3)	2.7 (6.4)	49.8 (28.3)	55.6 (40.5)	5.8 (13.9)	61.0 (30.7)	65.8 (32.9)	4.8 (12.2)	*P *= 0.83
CSF F_2_-IsoPs **(ng/mL)**	0.035 (0.005)	0.029 (0.009)	-0.006 (0.008)	0.023 (0.005)	0.022 (0.005)	-0.001 (0.005)	0.030 (0.008)	0.031 (0.013)	0.001 (0.009)	*P *= 0.08

CSF analyses are for 10 subjects in the placebo group, seven in the 2 gm/day group, and eight in the 4 gm/day group. Two subjects in the 4 gm/day group were lacking valid plasma Aβ_40 _levels and ADCS-ADL data was missing on one subject in each group. Aβ, amyloid beta; ADAS-Cog, Alzheimer's Disease Assessment Scale - Cognitive Subscale; ADCS-ADL, Alzheimer's Disease Cooperative Study - Activities of Daily Living; CSF, cerebrospinal fluid; NPI, Neuropsychiatric Inventory; p-tau, phosphorylated tau.

### Pharmacokinetic results

Using HPLC methods, levels of native curcumin were undetectable in any sample at any time point post-dose except for one sample four hours after a 4 gm dose (6 ng/mL). Therefore, LC/MS/MS was used as a more sensitive method to measure native curcumin and tetrahydrocurcumin and their glucuronidated metabolites. At the 24-week visit the baseline mean plasma concentrations of native curcumin and its free metabolite tetrahydrocurcumin were 2.67 ± 1.69 and 6.87 ± 4.91 ng/mL, respectively (< 10%CV). Three hours after medication administration the mean plasma concentrations of native curcumin and tetrahydrocurcumin were 7.76 ± 3.23 and 3.73 ± 2.0 ng/mL, respectively. The levels of glucuronidated curcumin and tetrahydrocurcumin were 96.05 ± 26 ng/mL and 298.2 ± 140.04 ng/mL. Plasma levels of curcumin were not detectable in placebo-treated subjects nor in persons prior to treatment with curcumin. Levels of native curcumin were undetectable in the CSF of placebo or curcumin-treated subjects.

## Discussion

In this 24-week double-blind placebo-controlled study with a 24-week extension, we examined the tolerability of 2 gm/day and 4 gm/day of Curcumin C3 Complex^® ^in persons with mild-to-moderate AD. A total of 4/35 (11.4%) subjects dropped out of the study while on curcumin, due to gastrointestinal side effects. Two subjects had black stools consistent with melena but in no subject was there unequivocal evidence of gastrointestinal hemorrhage or hemodynamic compromise. A subset of subjects underwent bleeding time assessments and no significant effect of curcumin was observed. The reason for the observed increased plasma glucose levels in persons treated with curcumin is unclear. These changes in hematocrit and blood glucose were relatively minor and values did not exceed the range of normal.

Following single doses of 2 gram and 4 gram doses of Curcumin C3 Complex^°^, we were unable to measure significant plasma levels of native curcumin based on the half maximal inhibitory concentration IC50's identified in Begum *et al. *[6]. Recent studies of curcumin suggest limited bioavailability [20], likely due to extensive metabolism in the gastrointestinal tract. In animal models, the diketone bridge of native curcumin was required for its plaque-reducing effects, presumably due to its requirement for amyloid-binding [6]. As an equivocal ameliorative peripheral effect on joint inflammation was suggested by our findings, the current results do not exclude a peripheral effect of glucuronidated curcuminoids, which were high, but as has been shown in mice [21], not likely to penetrate the blood brain barrier. However, we did not observe an effect on F_2_-IsoP levels that have been previously shown to be influenced by the administration of curcumin in a rat model of Aβ-induced injury [22] and by antioxidants in humans [23]. Alternative formulations of curcumin that are better absorbed are being studied [24] for their potential use in AD.

We were unable to demonstrate clinical or biochemical evidence of efficacy against AD. Considering the small sample size and short duration of this study, significant impact on clinical indices was not expected. Additionally, variability in the baseline disease severity may have masked effects, particularly if the intervention was effective in a subgroup (for example, persons with milder disease). However, there were non-significant trends for both MMSE score and the ADAS-Cog score to worsen slightly in the curcumin-treated groups relative to placebo. Though the small sample size and non-significance precludes definitive conclusions from this observation, it is possible that curcumin might exert a minor encephalopathic effect though the mechanism of this would be unknown.

A prior six-month study of curcumin in 34 persons with AD similarly failed to demonstrate an impact on clinical measures [25]. This study similarly found high levels of glucuronidated curcuminoids in plasma and a trend for increased Aβ40 with a 4 gram dose of curcumin was noted, possibly reflecting mobilization of Aβ40. However, we did not observe such an effect in our study and no effects on CSF AD biomarkers, including Aβ42, were found.

There have been relatively few clinical trials in which comprehensive biomarker data have been collected at six-month intervals. The data collected here show the baseline and 24-week variation in plasma Aβ, CSF Aβ_42_, CSF t-tau and p-tau_181 _and CSF isoprostanes (Table 2). Although the numbers are small these values may be of comparative interest for other trials.

Curcumin has anti-oxidant, anti-inflammatory and anti-amyloid effects *in vitro *and prior studies in animal AD models make it a promising avenue to pursue in human AD. There are many possible explanations for the results of our study including differences in the biology of rodent models of amyloidosis and human AD [26] and differences in the metabolism of curcumin between humans and rodents. It is unclear whether lack of a demonstrable benefit is due to problems with bioavailability of this specific formulation of curcumin or to inefficacy of curcumin as an intervention for AD. Further studies of other preparations of curcumin for AD are ongoing.

## Abbreviations

Aβ: amyloid beta; AchE-: acetylcholinesterase inhibitor; AD: Alzheimer's Disease; ADAS-Cog: Alzheimer's Disease Assessment Scale - Cognitive Subscale; ADCS-ADL: Alzheimer's Disease Cooperative Study - Activities of Daily Living; ANOVA: analysis of variance; APPsw: amyloid precursor protein Swedish mutation; APPsw/PS1dE9: amyloid precursor protein Swedish mutation/Presenilin 1 E9 deletion; APPsw: amyloid precursor protein Swedish mutation; CSF: cerebrospinal fluid; CV: coefficient of variance; F_2_-IsoPs: F2-isoprostanes; HPLC: high performance liquid chromatography; IL: interleukin; MMSE: Mini-Mental Status Examination; NPI: Neuropsychiatric Inventory; p-tau: phosphorylated tau; PBS: phosphate-buffered saline; SEM: standard error of the mean.

## Competing interests

SAF is a recipient of a grant from Veterans Affairs to study the efficacy of an alternative form of curcumin in persons at-risk for Alzheimer's Disease. The other authors report no competing interests.

## Authors' contributions

JMR led the study and implemented regulatory affairs, helped design the study, saw to its execution, lead the data analysis and interpretation, and prepared the initial draft of the manuscript and saw to its revisions. JB assisted with regulatory affairs and study conduct. SF assisted with study design, laboratory analyses and manuscript preparation. ET assisted with study conduct, laboratory analyses, data analysis and interpretation and manuscript preparation. VB advised on study design and on manuscript preparation. DDH performed laboratory analyses. LGA, VP, GM and ZV assisted with study conduct and manuscript preparation. GH and CS assisted with study design, data analysis and interpretation and manuscript preparation. TJM, KG and ANB performed laboratory analyses and assisted with manuscript preparation. JLC and DM assisted with study design, helped obtain funding and assisted with manuscript preparation. GMC helped with study design, study conduct, laboratory analyses and with manuscript preparation. All authors have read and approved the final manuscript.

## Acknowledgements

Most importantly, we would like to thank the participants of this study for their involvement. This study was supported by the John Douglas French Foundation and the Institute for the Study of Aging. The investigational product was provided by Sabinsa Corporation, Piscataway, NJ. Further support for this study came from the Sidell Kagan Foundation, the Shirley and Jack Goldberg Trust, Alzheimer's Disease Research Center Grant P50 AG-16570 and U01AG28583 (SAF) from the National Institute on Aging, General Clinical Research Centers Program M01-RR00865, a California Alzheimer's Disease Center grant, and the Easton Consortium for Alzheimer's Disease Drug Discovery and Biomarkers.

### Role of the Sponsors

Design and conduct of the study: The John Douglas French Foundation, Institute for he Study of Aging, Sidell Kagan Foundation, Jack Goldberg Trust, NIH GCRC M01-RR00865 grant, NIA P50 AG-16570.

Collection, management, analysis and interpretation of the data: California Alzheimer's Disease Center Grant, Jack Goldberg Trust, Sidell Kagan Foundation, NIH GCRC M01-RR00865 grant.

Preparation, review and approval of the manuscript: NIA P50 AG-16570.
